# [Retracted] Downregulation of miR-183 inhibits the growth of PANC-1 pancreatic cancer cells *in vitro *and *in vivo*, and increases chemosensitivity to 5-fluorouracil and gemcitabine 

**DOI:** 10.3892/etm.2026.13235

**Published:** 2026-07-07

**Authors:** Xiaoping Yang, Wei Wang, Xiong Zhang, Qi Zou, Lei Cai, Bo Yu

Exp Ther Med 17:1697–1705, 2019; DOI: 10.3892/etm.2018.7112

Following the publication of the above paper, it was drawn to the Editor’s attention by a concerned reader that certain of the flow cytometric data shown in Fig. 2A on p. 1701 were strikingly similar to data that subsequently appeared in the journal *Molecular Medicine Reports* in an article written by different authors at different research institutes, which itself has been retracted on account of sharing data with other unrelated papers. In addition, it was noted that there was a possible duplication of cell-cycle analysis data shown within Fig. 2B on p. 1701. Moreover, it was noted that the values along the *y*-axis scale bar for the tumor volumes (in mm^3^) in Fig. 3B appeared to be a factor of 10 lower than they ought to have been, i.e., so the scale should have read from 0-6,000 mm^3^ rather than from 0-600 mm^3^. Also in relation to this figure, the published tumor images seem inconsistent with the reported tumor volume data. Given that these various issues have come to light as being problematic with this article, the Editor of *Experimental and Therapeutic Medicine* has decided that this paper should be retracted from the Journal. The authors were asked for an explanation to account for these concerns, but the Editorial Office did not receive a reply. The Editor apologizes to the readership for any inconvenience caused.

